# A Crisis of Humanitarianism: Refugees at the Gates of Europe

**DOI:** 10.15171/ijhpm.2019.22

**Published:** 2019-04-22

**Authors:** Marianna Fotaki

**Affiliations:** Warwick Business School, University of Warwick, Coventry, UK.

**Keywords:** Rrefugee ‘Crisis’, European Union, Securitization, Healthcare, Human Rights

## Abstract

Having initially welcomed more than a million refugees and forced migrants into Europe between 2015 and 2016, the European Union’s (EU’s) policy has shifted toward externalising migration control to Turkey and Northern Africa. This goes against the spirit of international conventions aiming to protect vulnerable populations, yet there is widespread indifference toward those who remain stranded in Italy, Greece and bordering Mediterranean countries. Yet there are tens of thousands living in overcrowded reception facilities that have, in effect, turned into long-term detention centres with poor health and safety for those awaiting resettlement or asylum decisions. Disregard for humanitarian principles is predicated on radical inequality between lives that are worth living and protecting, and unworthy deaths that are unseen and unmarked by grieving. However, migration is on the rise due to natural and man-made disasters, and is becoming a global issue that concerns us all. We must therefore deal with it through collective political action that recognises refugees’ and forced migrants’ right to protection and ensures access to the health services they require.

## Introduction

In 2015, more than a million refugees and forced migrants, including children, women and men, crossed into the European Union (EU) by land and sea.^1^ Fleeing from war and persecution, the early waves of people arriving on the shores of the Mediterranean attracted the sympathy of receiving societies in various EU countries. Local communities in Greece and Italy, hand-in-hand with networks of volunteers from around the world, responded to their urgent needs, including health and safety,^2,3^ and many others helped them on their way to Germany and Sweden.^4,5^ Yet the initial welcome, epitomised in German Chancellor Angela Merkel’s statement “we can do it,”^6^ was quickly replaced by rising hostility toward the refugees and would-be migrants. Only a few months later, the Chancellor revised her policy, reaching an agreement with Turkey on behalf of the EU to hold back the arrivals in exchange for billions of Euros, free travel visas for its citizens, and a commitment from the EU to take the equivalent number of people directly from Turkish refugee camps.^7^ This marked an important shift toward securitisation, involving extreme politicisation of migration and its presentation as a security threat.^8^ Security policies are closely interwoven and entangled with humanitarian policies and transnational attempts to stabilise and enforce a specific, highly restrictive asylum system.^9^ This, I argue, signifies a departure from the principles of universal humanitarianism developed in Europe after the Second World War,^10^ and has led to a politically ambiguous and deeply paradoxical humanitarianism^11^ driven by limited moral considerations unsuited to addressing the impending increase in migration.^12^

Public debates and academic research centre around the actual or alleged impact of the incoming populations on their host societies’ economies, as well as on social and political development. Much less attention is being given to how the arriving refugees and forced migrants are coping with the trauma of the dramatic events that led to their flight, and with the aftermath of the traumatic journeys in which tens of thousands have lost their lives.^12^ There is insufficient recognition of the health needs of migrants, forced refugees and asylum seekers as a structural part of society, rather than as ‘external’ to health systems and other areas.^13^ The anti-migration rhetoric is becoming a permanent fixture of European politics,^14,15^ even though the mass arrival of more than 1.3 million people into the EU by sea in 2015 and 2016^16^ reduced dramatically to 120 000 in 2017 and to just over 60 000 in the first six months of 2018.^17^ In addition to the perception of refugees and migrants as threats to the economy and security, anxiety is often attributed to concerns surrounding globalisation and multiculturalism.^18^ However, anti-migration sentiments may be a form of anxiety displacement arising from the dislocation experienced by citizens of many developed European countries in economies with decreasing opportunities for meaningful employment and an increasingly privatised welfare state.^15^ In the aftermath of the global financial crisis, this dislocation has been exacerbated by retrenchment of the welfare state following the neoliberal shift in public policy over the past few decades. This is best reflected in the case of Greece, as the gateway to Europe in the recent refugee crisis, as well as in bordering Mediterranean countries such as Italy that receive refugees.

## Refugee Crisis or Refugees in Crisis?

Tens of thousands of people are currently in Greece, living in difficult conditions as they await resettlement, repatriation/deportation or asylum decisions.^19^ Many are living in overcrowded former military camps, abandoned factories and other disused public buildings such as summer camps and orphanages, with inadequate facilities to host them, and often without health and social services to meet even their basic needs. Addressing the health and housing needs of these people at short notice would have been financially and logistically challenging for any country^20^; but Greece’s own problems exacerbate these difficulties, as it is experiencing the longest and deepest recorded economic depression during peacetime.^21^ Its cash-starved public health system and bankrupt social security^22^ are unable to deal with its own citizens’ increasing needs, let alone those of the migrant populations now held in reception facilities (hot-spots) directed by the police and military in various mainland and island locations. Unlike Italy,^23^ Greece has had little experience of receiving and integrating refugees from diverse cultures.^22,24^ Even in Italy, “the reception system for asylum seekers and refugees, expanding to reach just over 180 000 places as of December 31, 2017, continues to be based, for the most part, on extraordinary reception structures in which services aimed at social inclusion are limited.”^25^

Refugees and forced migrants hope to rebuild their lives, but not necessarily in Greece or Italy given these countries’ domestic problems. For instance, Greece has a 23% overall unemployment rate, and youth unemployment reaching almost 50%,^26^ while many citizens in Italy are as marginalised as their migrant neighbours, living in informal settlements and engaging in informal occupations.^25^ Yet the refugees’ and forced migrants’ onward journeys to Northern European countries in search of a better future are made more haphazard and uncertain by current EU policies. Most refugees and migrants find themselves in legal limbo, subsisting in sub-human^27^ conditions in hot-spots in the Mediterranean that are, in effect, detention camps, with insufficient medical support^28^ and restricted movement (for instance, in Greece they are not allowed to leave the islands). Even reuniting with their families is fraught with increasing difficulty. Feeling desperate about being separated, people are often willing to put their lives in the hands of smuggler networks to reunite with their families across different countries, or even within the same country.^29^ Taken together, these factors exert extreme psychological pressure, putting people at increased risk of mental health breakdown^19,27^ and various psychosocial diseases. A recent study of 1293 refugees and forced migrants in three reception facilities in Greece found that most considered their access to legal information and assistance with asylum procedures to be poor to non-existent, and their uncertain status exacerbated their anxiety.^19^ Their various mental illness are caused mainly by external factors rather than by any prior mental health disorder.^27^ Overall, the newcomers’ health problems arise both from traumatic events in their own countries and the privations of their journeys, and from poor living conditions since their arrival, despite efforts by the Greek government to address cases of extreme vulnerability through emergency legislation and financial and logistical assistance from the EU. For instance, a list of people belonging to vulnerable groups is set out in relevant national legislation (see Box 1), giving them preference for accommodation outside camps with inadequate water supplies and poor sanitation, which offer no privacy or safety in overcrowded facilities.

**Box 1. Greek Law Offering Protection to the Vulnerable Refugees, Forced Migrants and Asylum Seekers**

According to Article 14(8) of Greek law L 4375/2016, vulnerable groups are: *(a)* unaccompanied minors, *(b)* people who have a disability or are suffering from an incurable or serious illness, *(c)* the elderly, *(d)* women in pregnancy or having recently given birth, *(e)* single parents with minor children, *(f)* victims of torture, rape or other serious forms of psychological, physical or sexual violence or exploitation, and people with a post-traumatic disorder, particularly survivors and relatives of victims of shipwrecks, and *(g)* victims of trafficking in human beings.^27,29^

This policy suggests that people with legally-defined vulnerabilities should be prioritised. However, such logic might be at odds with securing universal healthcare for all who need it that derives from a rights-based approach, as stipulated by the World Health Organization.^13^ In Greece and Sicily, entry points for arrivals into Europe, medical care provision varies significantly from one centre to another, with uneven access where it does exist. This is because many reception facilities are in out-of-town locations, and hot-spot managers lack information on services available.^23,24^ Access to the psychological help that many desperately require is even more scarce.^27^ Contrary to false alarms about the alleged health risk posed by refugees to receiving societies, it is they who face many risks to their health and wellbeing, as well their dignity. Legally-defined vulnerability based on different categories of need may therefore have the unintentional effect of promoting a new, restricted version of humanitarianism. Overall, such measures do not address refugees’ and forced migrants’ essential problems, and fail to ensure that these people have liveable lives and futures.^30^ For instance, even though the right to family life and protection of the family is enshrined in international human rights law, and is a shared value across cultures, the vulnerability policy forces families to continue to live apart after being separated during displacement, exposing them to the associated risks.^29^ The EU and its member states are failing to protect this right for refugees and migrants, as their policies and practices tear families apart. The challenges experienced by refugees and forced migrants at the EU frontier in Greece and Italy, including unsanitary and unsafe conditions often without healthcare, shelter, food and water,^25,27,28^ exemplify the need for collective solutions based on principles of universality rather than short-lived displays of solidarity through voluntary efforts.

## The Value and Values of Humanitarianism Re-envisioned?

The fate of refugees and forced migrants is now met mainly with indifference, or even enmity, even though the current numbers of people arriving into Europe hardly constitute a “migratory” or “refugee” crisis. This is not new: the dispossessed and refugees of the past often met with indifference and rejection by those who might help.^31^ Researchers have identified a range of social and individual factors, including public policies, discourses propagated by politicians and the media as well as prosocial orientation, that may facilitate or impede our willingness to receive refugees and migrants into our societies.^18^ Underlying these are complex psychosocial processes of categorising belonging and who counts as an outsider. These do not necessarily or exclusively involve rational evaluations and calculative thinking, but are often driven by unconscious fears and affective dynamics that are central to defining both who deserves help, and the level of help that individuals are willing to provide to them.^15^

International humanitarian law, enshrined in the 1951 Geneva Convention and the 1967 Protocol,^32^ sets out ratifying states’ obligations to protect refugees (civilian victims of war and armed conflict), with the intention of overcoming the randomness and unpredictability of such provision. Seeking to establish universal principles and values worth protecting in shielding refugees from harm aims to prevent situations where stateless people become, in Arendt’s words, “rightless”^33^ and are therefore exposed to every arbitrariness imaginable. I also suggest that the Convention recognises our universally-shared vulnerability and capacity for suffering, without instituting moral hierarchies or distinctions. This shift to humanitarianism originating in a rights-based approach is a recent invention with origins in medical ethics.^13^ Humanitarian medical providers, such as the Red Cross and Médecins sans Frontières, are playing an important role in this transformation. However, although protecting universal human values is now a potent force in our world,^11^ this situation may change. As philosopher Giorgio Agamben^34^ cautions, “the refugee is the sole category in which it is possible today to perceive the forms and limits of a political community to come.”

Arguably, the EU–Turkey deal exemplifies the potential directions and implications of such developments. This agreement^7^ may be in breach of the Geneva Convention’s non-refoulment rule (the forcible return of refugees or asylum seekers to countries where they are liable to be subjected to persecution), as Turkey falls short of the criteria for a safe first country in which asylum concepts can be applied. Human rights organisations report repeated shootings of Syrian refugees fleeing war at the Turkish border.^35,36^ As an article in *Der Spiegel* notes, fewer people are now drowning in the Aegean, as the number of boat crossings to Greece has decreased since the signing of the agreement; instead, refugees are now dying at the Turkish–Syrian border.^37^ In 390 of the 393 decisions issued by the Greek Appeals Committees concerning the repatriation/deportation of refugees and forced migrants, the requirements of national and EU law to consider Turkey a safe third country have not been fulfilled.^38^ Although Turkish citizens are not allowed to travel freely in the EU and relatively few migrants have been returned to or taken out of Turkey, there is little interest in the fate of the almost 60 000 people who have been left stranded in Greece following the EU–Turkey deal. European countries are turning their backs on their international obligations, as expressed in the EU’s recent policy of “externalising” migration controls to Turkey and Northern Africa, and there is evidence of the EU and member states targeting and criminalising defenders of the rights of people on the move.^39^ If continued, these changes may signal a move toward a new form of biopolitics, whereby worthy lives and suffering are made visible, while unworthy deaths are unseen and unmarked by grieving.^30^ Such biopolitics are predicated on the idea of radical inequality between sacred life on the one hand (eg, Western soldiers) and sacrificed life on the other (eg, local civilians).^40^

These developments call into question the principle of a universal, inalienable right to protection at a fundamental level, reinforcing the call for individual action in the face of states’ ostensible neglect but with no impetus for political change.^41^ Relying on individual and/or voluntary efforts may thus promote a type of humanitarianism that does not consider the causes of dispossession, but merely aims to build a temporary illusion of bridging the contradictions that give rise to global inequalities, and to “make the intolerableness of its injustices somewhat bearable” (p. xii).^11^ Yet humanitarianism, which focuses first and foremost on individual suffering, need not be incompatible with politics.^42,43^ Compassion and ethics of care are essential components of individual and social life, while both affect and care are political, as they are concerned with how we share this world with others.^44^ Migration is on the rise due to natural and man-made disasters that will become the defining problem of the 21st century.^12^ It requires collective political action as it concerns us all. We cannot address it effectively by reducing our acceptance of refugees and forced migrants or ignoring their essential health and welfare needs. Instead we must protect human rights by attaching equal value to all lives within and beyond the borders of nation states whenever their survival is at risk. In offering such protection, we recognise our dependence on others for our own survival as individuals and social beings.

## Ethical issues

Not applicable.

## Competing interests

Author declares that she has no competing interests.

## Author’s contribution

MF is the single author of the paper.
